# Atypical presentation and behavior of oculomotor schwannoma: case report and literature review

**DOI:** 10.1016/j.bas.2026.107471

**Published:** 2026-10-15

**Authors:** Vojsava Leka, Aida Agastra, Stela Dodaj, Mirel Grada, Klarisa Prenga, Arben Rroji, Eugen Enesi, Mentor Petrela

**Affiliations:** a Department of Neurosurgery and Interventional Neuroradiology, American Hospital 3, Tirana, Albania; b University Hospital Center Mother Teresa, Tirana, Albania

**Keywords:** Oculomotor nerve, Schwannoma, Recurrent ophthalmoplegia, Pediatric, Magnetic resonance imaging, Conservative management

## Abstract

**Introduction:**

Oculomotor nerve schwannomas are rare, particularly in children. Recurrent painful oculomotor palsy may resemble recurrent painful ophthalmoplegic neuropathy (RPON), creating diagnostic uncertainty when an enhancing lesion is present along the nerve.

**Research question:**

How should recurrent painful oculomotor palsy be interpreted in a child with a persistent enhancing cisternal lesion, and how can longitudinal observation inform individualized management?

**Materials and methods:**

We report a patient who presented at 13 years of age with painful right oculomotor palsy and a focal enhancing cisternal lesion. The diagnosis was a radiologically diagnosed oculomotor nerve schwannoma; histological confirmation was not obtained. A narrative literature review contextualizes the clinical course and management, with a descriptive comparison of 14 previously reported patients with recurrent or intermittent symptoms.

**Results:**

During the initial episode, ptosis resolved over approximately four weeks following corticosteroid treatment. A second episode in 2022 resolved spontaneously. At the September 2025 assessment, ptosis was absent, but right pupillary dysfunction persisted. MRI demonstrated no reported interval change over approximately six years. Clinical improvement was not accompanied by radiological regression.

**Discussion and conclusion:**

The persistent focal lesion and its anatomical relationship to the symptomatic nerve favored a radiological diagnosis of schwannoma, although localized inflammatory neuropathy could not be definitively excluded. Observation may be reasonable in selected patients with stable lesions, acceptable neurological function, and reliable follow-up. This single case does not establish the superiority or general safety of conservative management.

## Highlights

• Recurrent painful oculomotor palsy accompanied a persistent cisternal lesion.
• Ptosis improved while the enhancing lesion remained radiologically unchanged.
• MRI favored schwannoma, but histological confirmation was unavailable.
• The lesion remained radiologically stable during six years of observation.
• Observation requires individualized assessment and continued follow-up.

## 1 Introduction

Schwannomas are generally benign, slowly growing nerve sheath tumors. Oculomotor nerve schwannomas (ONS) are uncommon and have been described mainly in case reports and small series. Published reviews document both pediatric and adult presentations, with considerable variation in lesion location, clinical course, and management (Douglas et al., 2022; Muhammad and Niemelä, 2019; Yang et al., 2013; Ohata et al., 2006). Historical reports extend back to the autopsy description by Kovacs in 1927 (Kovacs, 1927).

Although progressive oculomotor palsy is a recognized presentation, recurrent or fluctuating deficits have also been described (Kawasaki, 1999; Murakami et al., 2005; Kim et al., 2015). In children, this clinical pattern warrants consideration of RPON. A persistent enhancing lesion along the nerve complicates the differential diagnosis because both neoplastic and inflammatory processes may cause enhancement. In the absence of tissue confirmation, the diagnosis of schwannoma remains radiological.

We report pediatric-onset recurrent painful right oculomotor palsy associated with a radiologically diagnosed cisternal ONS that remained stable during approximately six years of observation. The case contributes a longitudinal comparison of fluctuating neurological deficits and a persistent enhancing lesion; recurrent symptoms in ONS have been described previously.

## 2 Case description

In September 2019, a 13-year-old boy presented with isolated right oculomotor palsy, characterized by complete right upper eyelid ptosis, diplopia, and orbital pain of three weeks’ duration (Fig. 1). He reported no nausea, photophobia, or phonophobia. Examination revealed no clinical features of neurofibromatosis, and the family history was unremarkable.

**Fig. 1:**
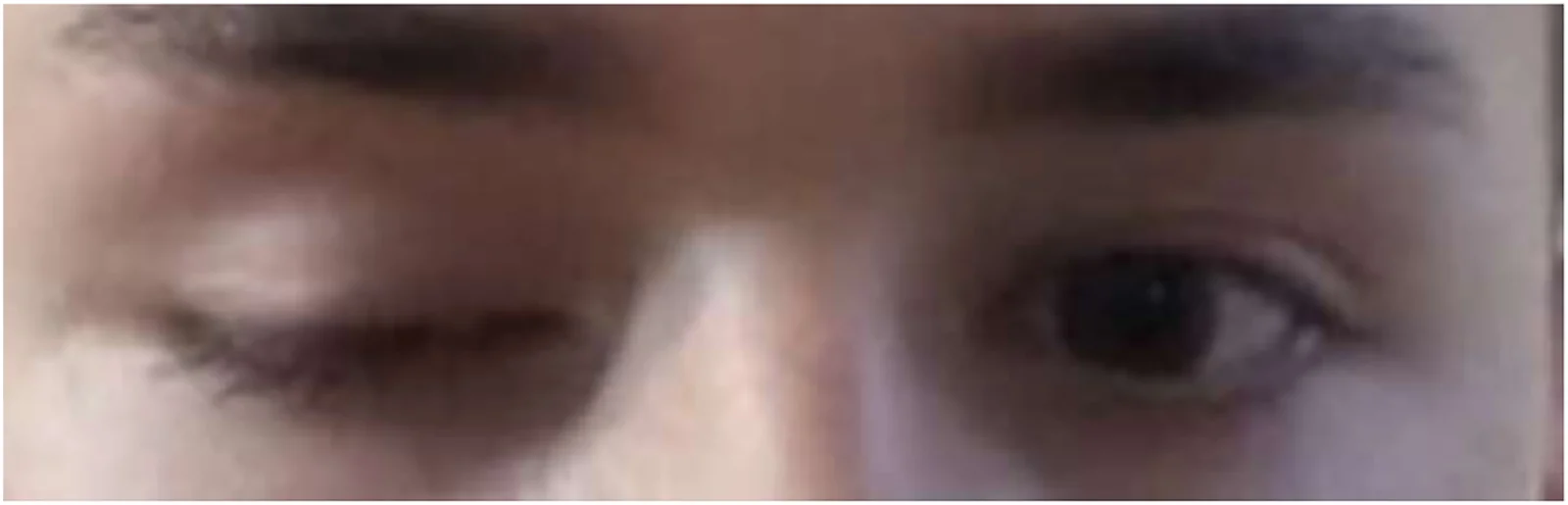
Clinical photograph obtained at the initial presentation in 2019, when the patient was 13 years old, demonstrating complete right upper eyelid ptosis.

Brain MRI demonstrated an extra-axial lesion with homogeneous contrast enhancement anterior to the right cerebral peduncle, along the expected course of the cisternal segment of the right oculomotor nerve (Fig. 2, Fig. 3). The anatomical location and imaging appearance supported a radiological diagnosis of cisternal ONS. Blood tests, immunological investigations, and cerebrospinal fluid analyses were reported as unremarkable. No biopsy or resection was performed, and the diagnosis was not histologically confirmed.

**Fig. 2:**
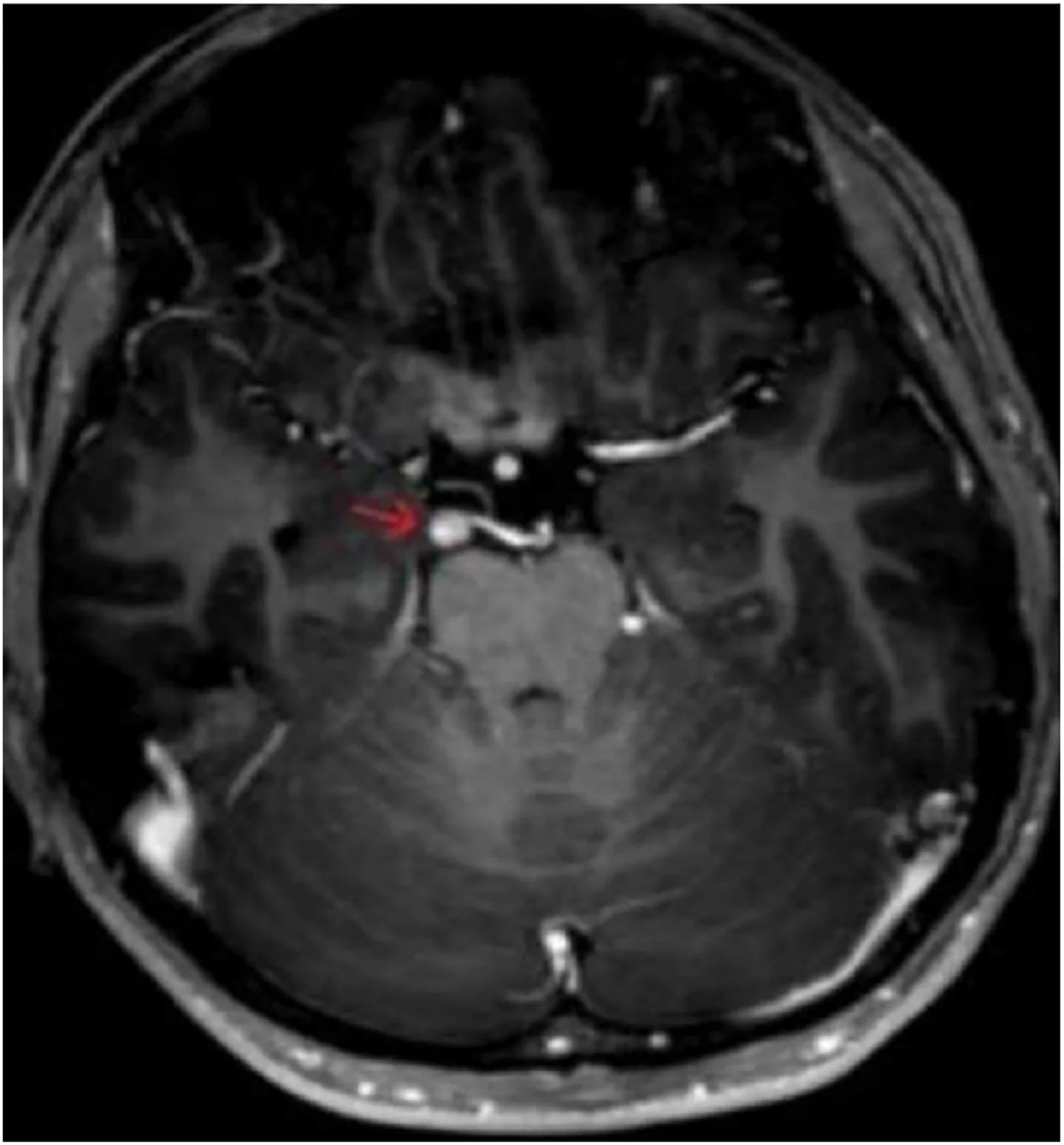
Axial gadolinium-enhanced T1-weighted MRI obtained at the initial presentation, demonstrating an enhancing lesion anterior to the right cerebral peduncle along the expected cisternal course of the oculomotor nerve.

**Fig. 3:**
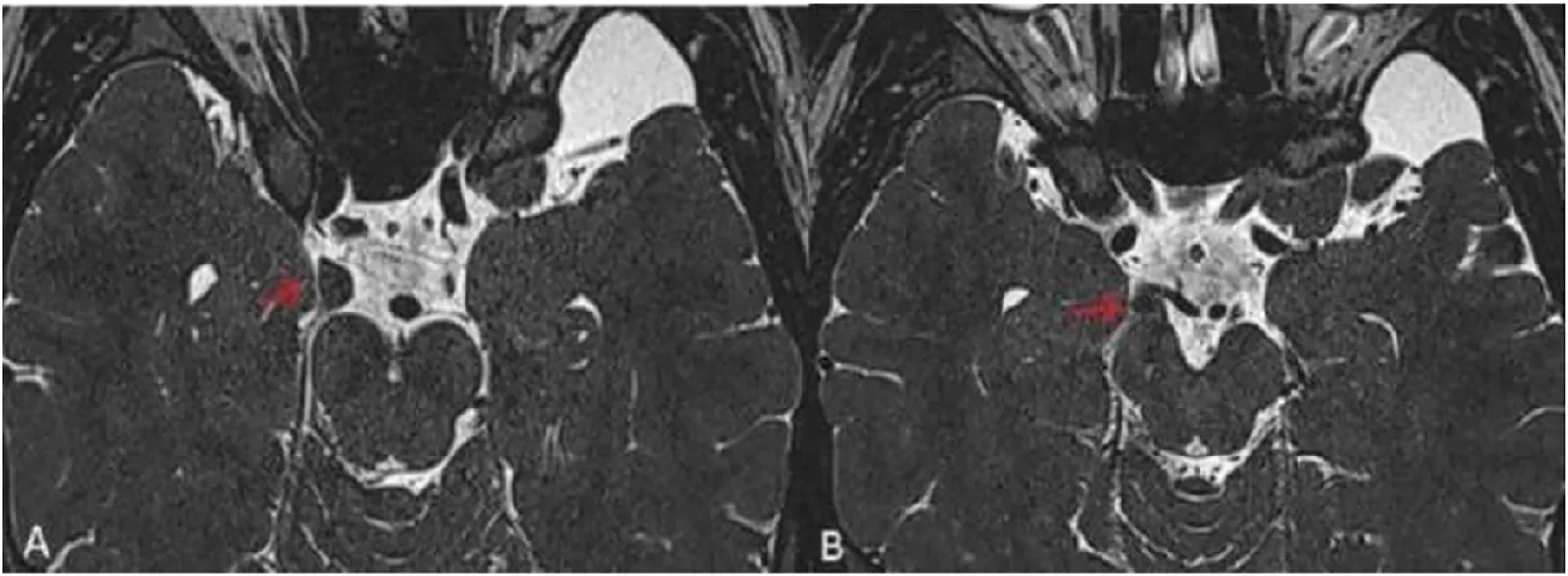
Axial T2-weighted DRIVE MRI obtained at the initial presentation, depicting the lesion in the region of the cisternal segment of the right oculomotor nerve.

The initial episode was treated with corticosteroids, and ptosis gradually resolved over approximately four weeks. The temporal association between treatment and recovery does not establish a treatment effect or confirm an inflammatory mechanism. After discussion with the family, observation with clinical and MRI follow-up was selected.

In 2022, the patient experienced a second episode of orbital pain and partial right upper eyelid ptosis (Fig. 4). The episode lasted approximately two weeks and resolved without treatment. Orbital pain and ptosis began simultaneously during both reported episodes.

**Fig. 4:**
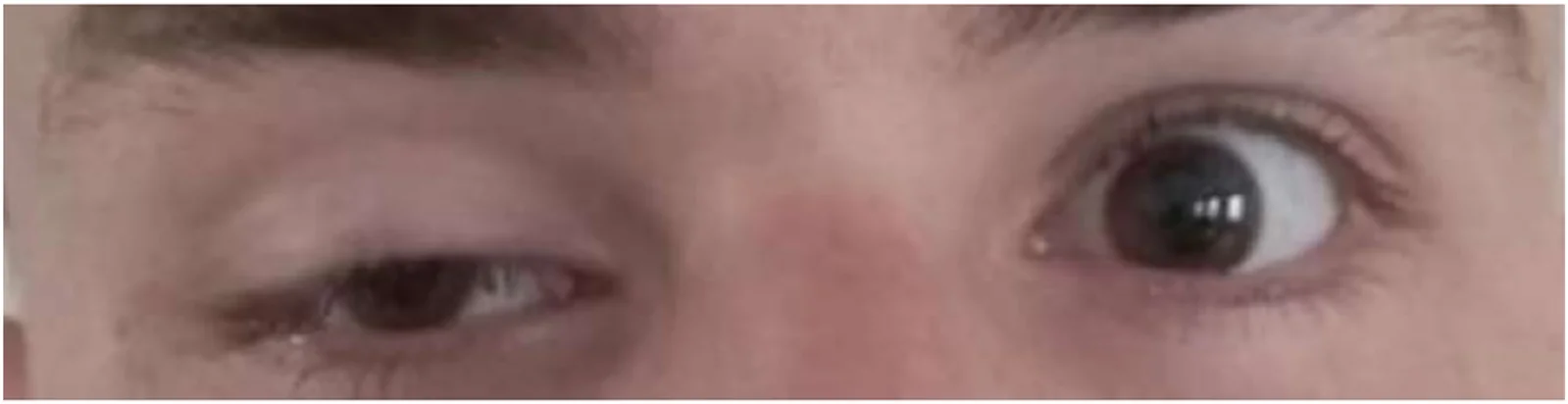
Clinical photograph obtained during the recurrent episode in 2022, demonstrating partial right upper eyelid ptosis.

At the September 2025 assessment, ptosis had resolved (Fig. 5), but anisocoria persisted: the right pupil measured 5 mm and the left pupil 3 mm. Direct and consensual light responses in the right pupil were delayed, whereas responses in the left pupil were intact. Follow-up MRI showed no reported interval change in lesion size or imaging characteristics relative to the initial study (Fig. 6, Fig. 7). Recovery of eyelid function therefore coexisted with residual pupillary dysfunction and a persistent enhancing lesion.

**Fig. 5:**
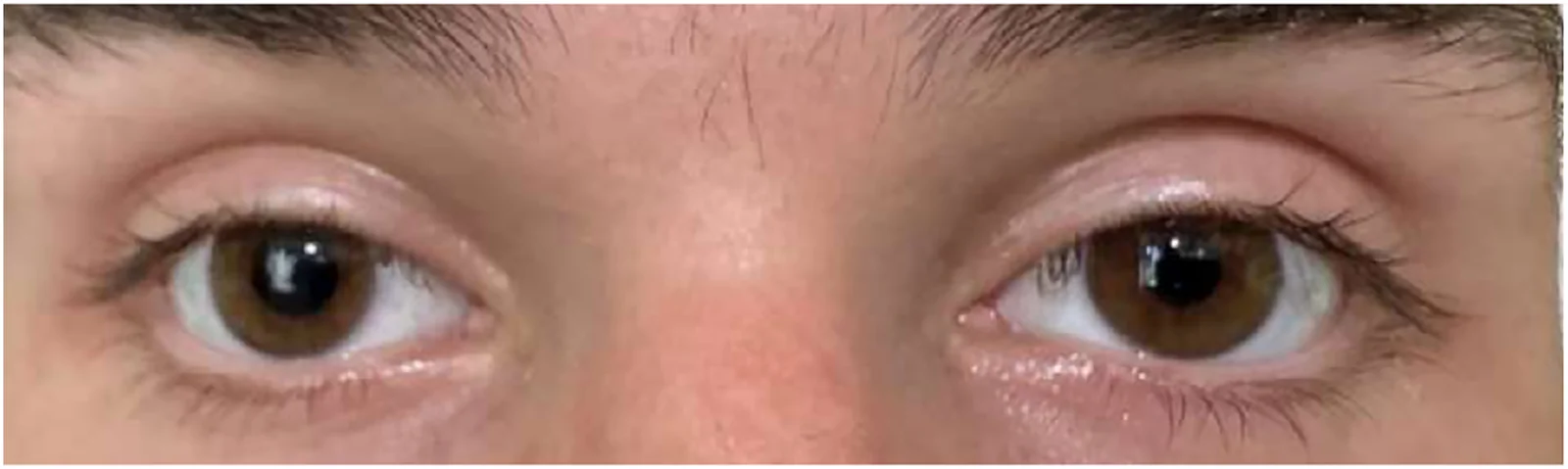
Clinical photograph obtained in 2025, demonstrating resolution of right upper eyelid ptosis. Residual right pupillary dysfunction was documented on examination.

**Fig. 6:**
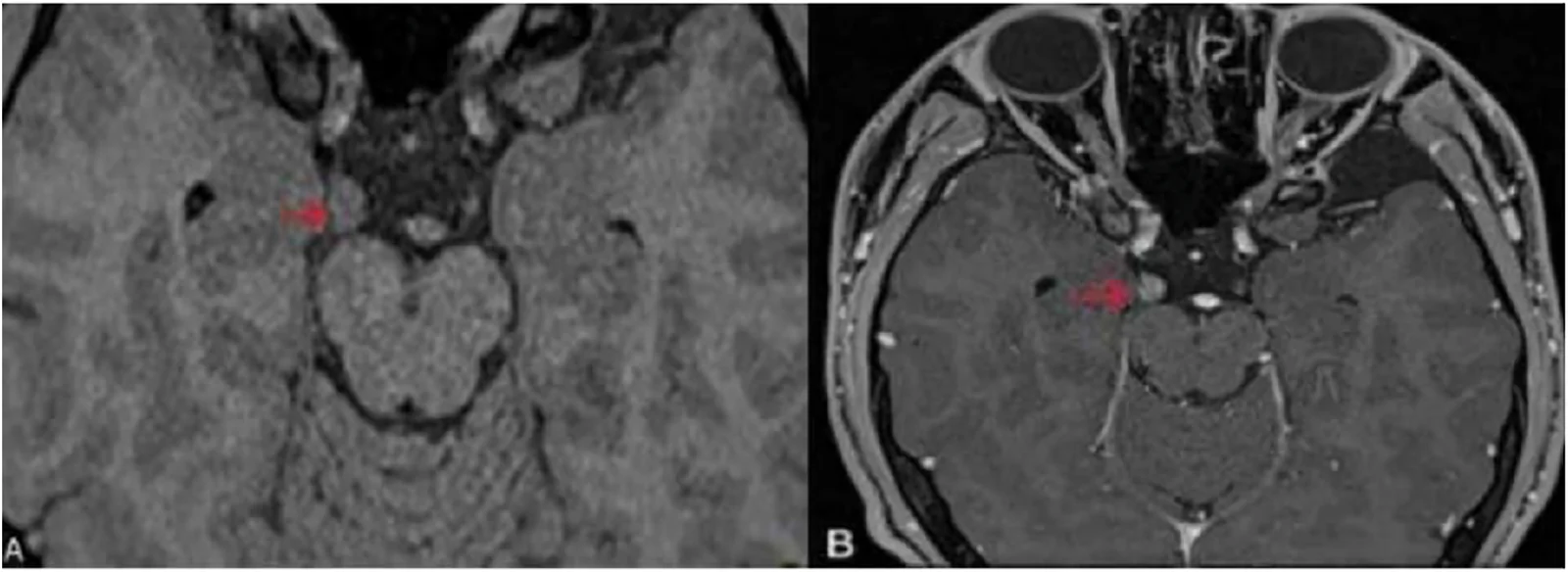
Axial T1-weighted MRI obtained before and after gadolinium administration in September 2025, demonstrating the persistent lesion without reported interval change.

**Fig. 7:**
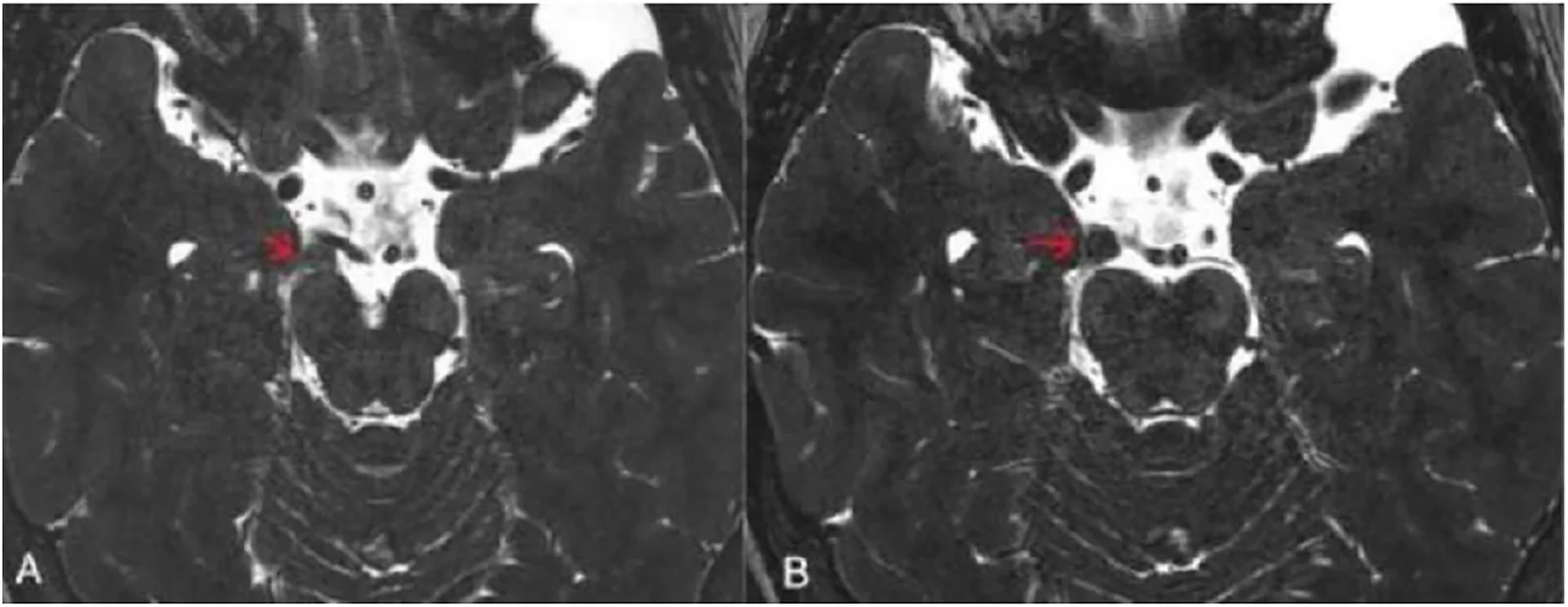
Axial T2-weighted SPACE MRI obtained in September 2025, depicting the persistent lesion in the region of the cisternal segment of the right oculomotor nerve.

## 3 Narrative literature review

This narrative review draws on historical reports and publications through 2024 identified using PubMed/MEDLINE, Google Scholar, ResearchGate, and relevant reference lists. It was not conducted as a systematic review, and the cases summarized should not be regarded as an exhaustive count of recurrent presentations or used to estimate their prevalence.

The descriptive comparison in Table 1 includes patients with recurrent or intermittent oculomotor dysfunction associated with a lesion reported as an oculomotor nerve schwannoma. Pain was recorded as a clinical variable rather than an inclusion criterion. Cases with exclusively progressive symptoms and reports without an associated oculomotor nerve lesion were excluded from this comparison. Patients were listed separately when a publication described more than one case. Imaging-based diagnoses were not treated as equivalent to histological confirmation.

**Table 1:** Previously reported patients with recurrent or intermittent oculomotor dysfunction associated with reported oculomotor nerve schwannoma.

Report or source	Age/sex	Lesion location	Pain	Episodes and age range as reported	Persistent MRI enhancement
Kawasaki (1999)	23/F	Cisternal	Yes	6 (7–23 years)	Not reported
Murakami et al. (2005)	11/F	Cisternal	Yes	2 (4–6 years)	60 months
Bisdorff et al. (2006)	14/F	Cisternal	Yes	>2 (4–14 years)	2 months
Riahi et al. (2014)	12/F	Cavernous sinus	Yes	3	Not reported
Kim et al. (2015)	52/M	Cisternal	Yes	Recurrent; count unspecified	12 months
Kim et al. (2015)	31/F	Cisternal	Yes	10 (26–31 years)	8 months
Shin et al. (2015)	41/F	Cisternal	Yes	3 (41–52 years)	13 months
Shin et al. (2015)	23/F	Cisternal	Yes	7 (7–30 years)	6 months
Shin et al. (2015)	43/M	Inferior division of CN III	No	2	10 months
Jibia et al. (2015)	13/F	Cisternal	Yes	Unspecified (7–13 years)	2 months
Abo-Shasha et al. (2018)	46/M	Anterior clinoid region	Yes	Unspecified (26–46 years)	13 years
Lee et al. (2018)	10/F	Cisternal	Yes	2 (2–10 years)	12 months
Petruzzelli et al. (2019)	16/M	Interpeduncular/perimesencephalic cistern	Yes	5 (6–16 years)	Not reported; lesion detected after a long symptom history
Mrabet et al. (2021)	18/F	Cisternal	Yes	4 (8–17 years)	12 months

CN III, third cranial nerve; F, female; M, male. Cases were collated from published reports and literature summaries (Kawasaki, 1999; Murakami et al., 2005; Kim et al., 2015; Mrabet et al., 2021; Petruzzelli et al., 2019). Ages and age ranges are reproduced as reported and may refer to different stages of the clinical course. The final column records the reported duration of persistent MRI enhancement, which is distinct from the duration of recurrent symptoms. “Not reported” indicates that this duration could not be established from the cited material. Pain was not required for inclusion. The table includes radiologically presumed lesions and should not be interpreted as a series of histologically confirmed schwannomas.

The oculomotor nerve has cisternal, cavernous, and intraorbital segments. Lesion location influences the clinical manifestations and the difficulty of imaging interpretation (Douglas et al., 2022; Lo et al., 2012). Early clinical reports and reviews described cisternal lesions and other anatomical presentations (Netuka and Beneš, 2003; Niazi and Boggan, 1994; Okamoto et al., 1985; Saetia et al., 2010). Cavernous sinus or parasellar lesions may be more difficult to attribute to an individual nerve, and imaging appearances can overlap with other tumors or vascular lesions (Chewning et al., 2008; Bentley et al., 2010).

Published reviews have expanded the descriptive evidence base, although management remains informed largely by heterogeneous case reports and small series (Douglas et al., 2022; Muhammad and Niemelä, 2019). A recent report illustrates the continued recognition of isolated cisternal lesions (Parida et al., 2024). Table 1 summarizes 14 previously reported patients, drawing on published case reports and literature summaries (Kawasaki, 1999; Murakami et al., 2005; Kim et al., 2015; Mrabet et al., 2021; Petruzzelli et al., 2019). These observations contextualize recurrent presentations but neither establish a distinct disease subtype nor demonstrate a shared histopathological diagnosis.

## 4 Discussion

### 4.1 Diagnostic certainty and the RPON differential

RPON is the principal clinical differential diagnosis because pediatric onset, recurrent ipsilateral orbital pain and oculomotor palsy, and recovery of ptosis between episodes are compatible with this disorder. However, this clinical pattern alone does not establish RPON. ICHD-3 also requires exclusion of an orbital, parasellar, or posterior fossa lesion by appropriate investigation (Headache Classification Committee of the International Headache Society, 2018). The persistent lesion in our patient therefore warrants assessment as a potential structural cause of the episodes.

The principal imaging features favoring a radiological diagnosis of ONS were anatomical concordance and longitudinal persistence. The extra-axial, homogeneously enhancing lesion lay along the expected cisternal course of the symptomatic right oculomotor nerve and remained unchanged over approximately six years despite clinical improvement. These imaging findings support a plausible anatomical relationship between the radiologically identified lesion and the recurrent ipsilateral oculomotor dysfunction. Enhancement alone cannot distinguish schwannoma from RPON, which may also produce nerve enhancement or thickening (Headache Classification Committee of the International Headache Society, 2018). The interpretation therefore rests on the combined anatomical and longitudinal findings rather than on enhancement during a single acute episode.

Published reports illustrate the diagnostic overlap between oculomotor nerve tumors and RPON. Kawasaki and Murakami et al. described schwannomas presenting with recurrent painful palsy (Kawasaki, 1999; Murakami et al., 2005), while Kim et al. reported persistent enhancing cisternal nodules in patients whose symptoms initially resembled RPON (Kim et al., 2015). These observations indicate that a relapsing–remitting course can occur in patients with reported or presumed oculomotor nerve tumors and does not, by itself, establish an inflammatory etiology.

Improvement after corticosteroid treatment does not exclude a presumed schwannoma. Although corticosteroids may benefit patients with RPON (Headache Classification Committee of the International Headache Society, 2018), Kim et al. also described symptomatic improvement after corticosteroid treatment in patients with radiologically presumed oculomotor schwannomas whose enhancing nodules persisted (Kim et al., 2015). The temporal association between treatment and recovery in our patient therefore does not establish an inflammatory etiology or a treatment effect. Spontaneous resolution of the subsequent untreated episode further limits causal attribution of the initial recovery to corticosteroids. In both episodes, improvement in ptosis must be distinguished from radiological regression, which was not observed.

Residual right pupillary dysfunction in 2025 indicates incomplete neurological recovery despite resolution of ptosis. Although anatomically concordant with involvement of the same nerve, this finding is not specific for schwannoma, because persistent deficits may also occur in RPON (Kim et al., 2015). In RPON, headache may precede ophthalmoplegia by up to 14 days (Murakami et al., 2005; Headache Classification Committee of the International Headache Society, 2018), whereas orbital pain and ptosis began simultaneously during both episodes in this patient. This temporal pattern is clinically relevant but does not independently exclude RPON. Similarly, the absence of migrainous features does not exclude RPON, and the time to resolution of enhancement should not be used as an absolute diagnostic threshold.

The focal lesion along the affected nerve and its persistence during clinical remission favor a structural nerve lesion over an unqualified diagnosis of primary RPON. Nevertheless, the attribution to schwannoma remains radiological. Approximately six years of stability supports an indolent process but does not establish histological identity. Unremarkable laboratory and cerebrospinal fluid findings also do not definitively exclude a localized inflammatory neuropathy. We therefore retain the working diagnosis of a radiologically diagnosed cisternal ONS, acknowledge inflammatory neuropathy and other indolent lesions in the differential diagnosis, and consider continued surveillance necessary.

### 4.2 Other diagnostic considerations

Tolosa–Hunt syndrome is relevant to painful ophthalmoplegia, particularly when imaging demonstrates a cavernous sinus or orbital inflammatory process (Lo et al., 2012; Saetia et al., 2010). In this case, the described abnormality was cisternal and anterior to the cerebral peduncle. This localization favors a lesion affecting the cisternal oculomotor nerve over a typical cavernous sinus process. Corticosteroid-associated improvement alone does not establish Tolosa–Hunt syndrome.

Aneurysmal compression is another important differential diagnosis in painful oculomotor palsy, particularly when the pupil is involved. Conversely, schwannoma may mimic a vascular lesion on imaging, as illustrated by Chewning et al. (2008). Exclusion of a vascular cause requires appropriate neuroradiological assessment and cannot be inferred from clinical recovery; vascular imaging should complement MRI when indicated (Lo et al., 2012). Detailed vascular imaging findings were not available for this report, so angiographic exclusion of an aneurysm cannot be asserted.

Other extra-axial lesions, including meningioma, may be considered according to location and morphology. Parasellar and cavernous sinus ONS can create particular diagnostic difficulties (Douglas et al., 2022; Bentley et al., 2010). In the present case, the reported relationship to the cisternal nerve course supported the working diagnosis, while the absence of tissue diagnosis limits exclusion of other indolent lesions.

### 4.3 Clinical contribution and management

Recurrent oculomotor dysfunction has been described previously in patients with reported or presumed nerve tumors, including painful and painless presentations (Kawasaki, 1999; Murakami et al., 2005; Kim et al., 2015). The present case contributes a longitudinal observation of pediatric-onset painful episodes, recovery of ptosis, residual pupillary dysfunction, and a persistent enhancing lesion over approximately six years. The distinction between neurological recovery and lesion behavior is central: clinical improvement was not accompanied by radiological regression.

Observation was selected after discussion with the family, and the lesion remained radiologically stable during follow-up. This course supports consideration of observation in selected patients with stable lesions, acceptable neurological function, and reliable access to continued assessment. It does not establish that observation is preferable to intervention in pediatric patients or in patients with presumed ONS generally.

Surgical intervention may provide tissue diagnosis and address growth or compression, but resection can impair oculomotor function (Muhammad and Niemelä, 2019). Radiosurgery is another reported management option, although its suitability depends on lesion characteristics and the clinical context (Douglas et al., 2022; Muhammad and Niemelä, 2019). Neither surgical nor radiosurgical outcomes can be compared with observation on the basis of this case. A management decision should consider diagnostic confidence, documented growth, neurological progression, lesion location, patient age, and the feasibility of surveillance.

Continued clinical and MRI surveillance remains appropriate, as stability during the reported follow-up does not establish long-term stability.

New or worsening neurological deficits, lesion growth, or changes in imaging characteristics would prompt reassessment of the diagnosis and management plan. An optimal surveillance interval cannot be determined from this case.

### 4.4 Limitations

This report describes a single patient without histological confirmation and cannot establish a causal relationship between the lesion and the recurrent symptoms. Quantitative lesion measurements, a standardized surveillance schedule, and detailed vascular imaging findings were not available for this report. Recovery from the first episode followed corticosteroid treatment, whereas recovery from the second was spontaneous; therefore, the therapeutic benefit of corticosteroids cannot be established.

The narrative review is subject to selection and publication bias, and the historical cases differ in diagnostic certainty, reporting detail, and follow-up duration. These limitations preclude general management recommendations or a reliable estimate of the frequency of recurrent presentations.

## 5 Conclusion

A persistent focal enhancing lesion along the oculomotor nerve should prompt consideration of a radiologically diagnosed schwannoma in patients with recurrent painful oculomotor palsy. Clinical recovery, including improvement after corticosteroid treatment, neither establishes RPON nor demonstrates lesion regression. In this patient, observation was associated with approximately six years of radiological stability, although pupillary dysfunction persisted. Observation may be reasonable in selected patients with stable lesions, acceptable neurological function, and reliable long-term follow-up. The absence of histological confirmation and the limitations of a single case preclude broader conclusions about diagnostic certainty or comparative treatment outcomes.

## Author contributions

Conceptualization: Mentor Petrela. Writing – original draft: Aida Agastra, Vojsava Leka, Mentor Petrela. Formal analysis: Aida Agastra, Vojsava Leka, Mentor Petrela. Methodology: Eugen Enesi, Arben Rroji, Mentor Petrela. Writing – review and editing: Mentor Petrela, Vojsava Leka, Aida Agastra, Klarisa Prenga, Stela Dodaj. Data curation: Vojsava Leka, Stela Dodaj, Mirel Grada. Supervision: Mentor Petrela.

## Ethics declaration

Written informed consent to take part in the study and to publish the article has been obtained from all participants or their legal representatives. The privacy rights of participants have been observed.

This study was performed in compliance with relevant laws, regulatory frameworks and guidelines where the research took place. Ethics committee approval was not required under relevant laws and institutional guidelines. This manuscript presents a single-patient retrospective case report based on clinical and radiological findings obtained during routine medical care at American Hospital 3, Tirana, Albania. No experimental interventions or additional research-related procedures were performed. Formal ethics committee approval was not required for this type of case report under the applicable institutional policies. Written informed consent for publication of the clinical information and accompanying images was obtained. Patient confidentiality and privacy were maintained throughout the preparation of the manuscript.

This research follows the CARE guidelines and the CARE checklist.

## Data availability

The data supporting this case report are available from the corresponding author upon reasonable request.

## Consent for publication

Informed consent was obtained from the patient for publication.

## Declaration of generative AI and AI-assisted technologies

During the preparation of this work, the authors used ChatGPT to assist with language editing and the organization of manuscript revisions. The authors reviewed and edited the resulting text and take full responsibility for the content of the manuscript.

## Funding

No external funding was received.

## Declaration of competing interests

The authors declare no competing interests.

## References

[bib1] Bentley J.N., Prabhu V., Bruner H. (2010). Large oculomotor schwannoma presenting as a parasellar mass: case report and literature review. Surg. Neurol. Int..

[bib2] Chewning R.H., Sasson A.D., Jordan L.C., Tamargo R.J., Gailloud P. (2008). Acute third cranial nerve palsy from a third cranial nerve schwannoma presenting as a saccular aneurysm on three-dimensional computed tomography angiography: case illustration. J. Neurosurg..

[bib3] Douglas V.P., Flores C., Douglas K.A., Strominger M.B., Kasper E., Torun N. (2022). Oculomotor nerve schwannoma: case series and literature review. Surv. Ophthalmol..

[bib4] Headache Classification Committee of the International Headache Society (2018). The international classification of headache disorders, 3rd edition. Cephalalgia.

[bib5] Kawasaki A. (1999). Oculomotor nerve schwannoma associated with ophthalmoplegic migraine. Am. J. Ophthalmol..

[bib6] Kim R., Kim J.H., Kim E., Yang H.K., Hwang J.M., Kim J.S. (2015). Oculomotor nerve tumors masquerading as recurrent painful ophthalmoplegic neuropathy: report of two cases and review of the literature. Cephalalgia.

[bib7] Kovacs W. (1927). Über ein solitäres Neurinom des Nervus oculomotorius. Zentralbl Allg Pathol.

[bib8] Lo C.P., Huang C.F., Hsu C.C. (2012). Neuroimaging of isolated and non-isolated third nerve palsies. Br. J. Radiol..

[bib9] Mrabet S., Nasri A., Kessentini N., Ben Djebara M., Gargouri-Berrechid A., Kacem I. (2021). Recurrent painful ophthalmoplegic neuropathy revealing oculomotor nerve schwannoma. Tunis. Med..

[bib10] Muhammad S., Niemelä M. (2019). Management of oculomotor nerve schwannoma: systematic review of literature and illustrative case. Surg. Neurol. Int..

[bib11] Murakami T., Funatsuka M., Komine M. (2005). Oculomotor nerve schwannoma mimicking ophthalmoplegic migraine. Neuropediatrics.

[bib12] Netuka D., Beneš V. (2003). Oculomotor nerve schwannoma. Br. J. Neurosurg..

[bib13] Niazi W., Boggan J.E. (1994). Schwannoma of extraocular nerves: survey of literature and case report of an isolated third nerve schwannoma. Skull Base Surg..

[bib14] Ohata K., Takami T., Goto T., Ishibashi K. (2006). Schwannoma of the oculomotor nerve. Neurol. India.

[bib15] Okamoto S., Handa H., Yamashita J. (1985). Neurinoma of the oculomotor nerve. Surg. Neurol..

[bib16] Parida S., Sachdeva V., Warkad V.U. (2024). Isolated cisternal third cranial nerve schwannoma: an insight into a clinico-radiological features. BMJ Case Rep..

[bib17] Petruzzelli M.G., Margari M., Furente F., Costanza M.C., Legrottaglie A.R., Dicuonzo F. (2019). Recurrent painful ophthalmoplegic neuropathy and oculomotor nerve schwannoma: a pediatric case report with long-term MRI follow-up and literature review. Pain Res. Manag..

[bib18] Saetia K., Valavanis A., Mendola C., Schaller K. (2010). Schwannoma of the oculomotor nerve: case report and review of the literature. Acta Neurochir.(Wien).

[bib19] Yang S.S. (2013). Pediatric isolated oculomotor nerve schwannoma: a new case report and literature review. Pediatr. Neurol..

