# Supplementary material for: Atypical presentation and behavior of oculomotor schwannoma: case report and literature review
Source: Brain Spine. 2026 Oct 15;6:107471. doi: 10.1016/j.bas.2026.107471 (PMC13695105; doi:10.1016/j.bas.2026.107471)
Supplement: Supplementary file 1 [file mmc1.docx]

Structured Literature Review

Atypical presentation and behavior of oculomotor schwannoma: case report and literature review

Article reference: BAS_BAS-D-26-00153 | Production reference BAS 107471

This literature review draws on historical reports and publications through 2024 identified using PubMed/MEDLINE, Google Scholar, ResearchGate, and relevant reference lists. It was not conducted as a systematic review, and the cases summarized should not be regarded as an exhaustive count of recurrent presentations or used to estimate their prevalence.

The descriptive comparison in Table 1 includes patients with recurrent or intermittent oculomotor dysfunction associated with a lesion reported as an oculomotor nerve schwannoma. Pain was recorded as a clinical variable rather than an inclusion criterion. Cases with exclusively progressive symptoms and reports without an associated oculomotor nerve lesion were excluded from this comparison. Patients were listed separately when a publication described more than one case. Imaging-based diagnoses were not treated as equivalent to histological confirmation.

The oculomotor nerve has cisternal, cavernous, and intraorbital segments. Lesion location influences the clinical manifestations and the difficulty of imaging interpretation [1,3]. Early clinical reports and reviews described cisternal lesions and other anatomical presentations [6,11,12,15]. Cavernous sinus or parasellar lesions may be more difficult to attribute to an individual nerve, and imaging appearances can overlap with other tumors or vascular lesions [13,14].

Published reviews have expanded the descriptive evidence base, although management remains informed largely by heterogeneous case reports and small series [1,4]. A recent report illustrates the continued recognition of isolated cisternal lesions [10]. Table 1 summarizes 14 previously reported patients, drawing on published case reports and literature summaries [7,9,17–19]. These observations contextualize recurrent presentations but neither establish a distinct disease subtype nor demonstrate a shared histopathological diagnosis.

# Table 1

Previously reported patients with recurrent or intermittent oculomotor dysfunction associated with reported oculomotor nerve schwannoma

| Report or source | Age/sex | Lesion location | Pain | Episodes and age range as reported | Persistent MRI enhancement |
| --- | --- | --- | --- | --- | --- |
| Kawasaki (1999) | 23/F | Cisternal | Yes | 6 (7–23 years) | Not reported |
| Murakami et al. (2005) | 11/F | Cisternal | Yes | 2 (4–6 years) | 60 months |
| Bisdorff et al. (2006) | 14/F | Cisternal | Yes | >2 (4–14 years) | 2 months |
| Riahi et al. (2014) | 12/F | Cavernous sinus | Yes | 3 | Not reported |
| Kim et al. (2015) | 52/M | Cisternal | Yes | Recurrent; count unspecified | 12 months |
| Kim et al. (2015) | 31/F | Cisternal | Yes | 10 (26–31 years) | 8 months |
| Shin et al. (2015) | 41/F | Cisternal | Yes | 3 (41–52 years) | 13 months |
| Shin et al. (2015) | 23/F | Cisternal | Yes | 7 (7–30 years) | 6 months |
| Shin et al. (2015) | 43/M | Inferior division of CN III | No | 2 | 10 months |
| Jibia et al. (2015) | 13/F | Cisternal | Yes | Unspecified (7–13 years) | 2 months |
| Abo-Shasha et al. (2018) | 46/M | Anterior clinoid region | Yes | Unspecified (26–46 years) | 13 years |
| Lee et al. (2018) | 10/F | Cisternal | Yes | 2 (2–10 years) | 12 months |
| Petruzzelli et al. (2019) [19] | 16/M | Interpeduncular / perimesencephalic cistern | Yes | 5 (6–16 years) | Not reported; lesion detected after a long symptom history |
| Mrabet et al. (2021) [18] | 18/F | Cisternal | Yes | 4 (8–17 years) | 12 months |

CN III, third cranial nerve; F, female; M, male. Cases were collated from published reports and literature summaries [7,9,17–19]. Ages and age ranges are reproduced as reported and may refer to different stages of the clinical course. The final column records the reported duration of persistent MRI enhancement, which is distinct from the duration of recurrent symptoms. “Not reported” indicates that this duration could not be established from the cited material. Pain was not required for inclusion. The table includes radiologically presumed lesions and should not be interpreted as a series of histologically confirmed schwannomas.

# Discussion

## Diagnostic certainty and the RPON differential

RPON is the principal clinical differential diagnosis because pediatric onset, recurrent ipsilateral orbital pain and oculomotor palsy, and recovery of ptosis between episodes are compatible with this disorder. However, this clinical pattern alone does not establish RPON. ICHD-3 also requires exclusion of an orbital, parasellar, or posterior fossa lesion by appropriate investigation [16]. The persistent lesion in our patient therefore warrants assessment as a potential structural cause of the episodes.

The principal imaging features favoring a radiological diagnosis of ONS were anatomical concordance and longitudinal persistence. The extra-axial, homogeneously enhancing lesion lay along the expected cisternal course of the symptomatic right oculomotor nerve and remained unchanged over approximately six years despite clinical improvement. These imaging findings support a plausible anatomical relationship between the radiologically identified lesion and the recurrent ipsilateral oculomotor dysfunction. Enhancement alone cannot distinguish schwannoma from RPON, which may also produce nerve enhancement or thickening [16]. The interpretation therefore rests on the combined anatomical and longitudinal findings rather than on enhancement during a single acute episode.

Published reports illustrate the diagnostic overlap between oculomotor nerve tumors and RPON. Kawasaki and Murakami et al. described schwannomas presenting with recurrent painful palsy [7,9], while Kim et al. reported persistent enhancing cisternal nodules in patients whose symptoms initially resembled RPON [17]. These observations indicate that a relapsing–remitting course can occur in patients with reported or presumed oculomotor nerve tumors and does not, by itself, establish an inflammatory etiology.

Improvement after corticosteroid treatment does not exclude a presumed schwannoma. Although corticosteroids may benefit patients with RPON [16], Kim et al. also described symptomatic improvement after corticosteroid treatment in patients with radiologically presumed oculomotor schwannomas whose enhancing nodules persisted [17]. The temporal association between treatment and recovery in our patient therefore does not establish an inflammatory etiology or a treatment effect. Spontaneous resolution of the subsequent untreated episode further limits causal attribution of the initial recovery to corticosteroids. In both episodes, improvement in ptosis must be distinguished from radiological regression, which was not observed.

Residual right pupillary dysfunction in 2025 indicates incomplete neurological recovery despite resolution of ptosis. Although anatomically concordant with involvement of the same nerve, this finding is not specific for schwannoma, because persistent deficits may also occur in RPON [17]. In RPON, headache may precede ophthalmoplegia by up to 14 days [9,16], whereas orbital pain and ptosis began simultaneously during both episodes in this patient. This temporal pattern is clinically relevant but does not independently exclude RPON. Similarly, the absence of migrainous features does not exclude RPON, and the time to resolution of enhancement should not be used as an absolute diagnostic threshold.

The focal lesion along the affected nerve and its persistence during clinical remission favor a structural nerve lesion over an unqualified diagnosis of primary RPON. Nevertheless, the attribution to schwannoma remains radiological. Approximately six years of stability supports an indolent process but does not establish histological identity. Unremarkable laboratory and cerebrospinal fluid findings also do not definitively exclude a localized inflammatory neuropathy. We therefore retain the working diagnosis of a radiologically diagnosed cisternal ONS, acknowledge inflammatory neuropathy and other indolent lesions in the differential diagnosis, and consider continued surveillance necessary.

## Other diagnostic considerations

Tolosa–Hunt syndrome is relevant to painful ophthalmoplegia, particularly when imaging demonstrates a cavernous sinus or orbital inflammatory process [3,15]. In this case, the described abnormality was cisternal and anterior to the cerebral peduncle. This localization favors a lesion affecting the cisternal oculomotor nerve over a typical cavernous sinus process. Corticosteroid-associated improvement alone does not establish Tolosa–Hunt syndrome.

Aneurysmal compression is another important differential diagnosis in painful oculomotor palsy, particularly when the pupil is involved. Conversely, schwannoma may mimic a vascular lesion on imaging, as illustrated by Chewning et al. [13]. Exclusion of a vascular cause requires appropriate neuroradiological assessment and cannot be inferred from clinical recovery; vascular imaging should complement MRI when indicated [3]. Detailed vascular imaging findings were not available for this report, so angiographic exclusion of an aneurysm cannot be asserted.

Other extra-axial lesions, including meningioma, may be considered according to location and morphology. Parasellar and cavernous sinus ONS can create particular diagnostic difficulties [1,14]. In the present case, the reported relationship to the cisternal nerve course supported the working diagnosis, while the absence of tissue diagnosis limits exclusion of other indolent lesions.

## Clinical contribution and management

Recurrent oculomotor dysfunction has been described previously in patients with reported or presumed nerve tumors, including painful and painless presentations [7,9,17]. The present case contributes a longitudinal observation of pediatric-onset painful episodes, recovery of ptosis, residual pupillary dysfunction, and a persistent enhancing lesion over approximately six years. The distinction between neurological recovery and lesion behavior is central: clinical improvement was not accompanied by radiological regression.

Observation was selected after discussion with the family, and the lesion remained radiologically stable during follow-up. This course supports consideration of observation in selected patients with stable lesions, acceptable neurological function, and reliable access to continued assessment. It does not establish that observation is preferable to intervention in pediatric patients or in patients with presumed ONS generally.

Surgical intervention may provide tissue diagnosis and address growth or compression, but resection can impair oculomotor function [4]. Radiosurgery is another reported management option, although its suitability depends on lesion characteristics and the clinical context [1,4]. Neither surgical nor radiosurgical outcomes can be compared with observation on the basis of this case. A management decision should consider diagnostic confidence, documented growth, neurological progression, lesion location, patient age, and the feasibility of surveillance.

Continued clinical and MRI surveillance remains appropriate, as stability during the reported follow-up does not establish long-term stability.

New or worsening neurological deficits, lesion growth, or changes in imaging characteristics would prompt reassessment of the diagnosis and management plan. An optimal surveillance interval cannot be determined from this case.

## Limitations

This report describes a single patient without histological confirmation and cannot establish a causal relationship between the lesion and the recurrent symptoms. Quantitative lesion measurements, a standardized surveillance schedule, and detailed vascular imaging findings were not available for this report. Recovery from the first episode followed corticosteroid treatment, whereas recovery from the second was spontaneous; therefore, the therapeutic benefit of corticosteroids cannot be established.

The narrative review is subject to selection and publication bias, and the historical cases differ in diagnostic certainty, reporting detail, and follow-up duration. These limitations preclude general management recommendations or a reliable estimate of the frequency of recurrent presentations.

# Conclusion

A persistent focal enhancing lesion along the oculomotor nerve should prompt consideration of a radiologically diagnosed schwannoma in patients with recurrent painful oculomotor palsy. Clinical recovery, including improvement after corticosteroid treatment, neither establishes RPON nor demonstrates lesion regression. In this patient, observation was associated with approximately six years of radiological stability, although pupillary dysfunction persisted. Observation may be reasonable in selected patients with stable lesions, acceptable neurological function, and reliable long-term follow-up. The absence of histological confirmation and the limitations of a single case preclude broader conclusions about diagnostic certainty or comparative treatment outcomes.

# References

1. Douglas VP, Flores C, Douglas KA, Strominger MB, Kasper E, Torun N. Oculomotor nerve schwannoma: case series and literature review. Surv Ophthalmol. 2022;67(4):1160–1174. doi:10.1016/j.survophthal.2021.11.008.

2. Kovacs W. Über ein solitäres Neurinom des Nervus oculomotorius. Zentralbl Allg Pathol. 1927;40:518–522.

3. Lo CP, Huang CF, Hsu CC, et al. Neuroimaging of isolated and non-isolated third nerve palsies. Br J Radiol. 2012;85(1012):460–467. doi:10.1259/bjr/38090653.

4. Muhammad S, Niemelä M. Management of oculomotor nerve schwannoma: systematic review of literature and illustrative case. Surg Neurol Int. 2019;10:40. doi:10.25259/SNI-75-2019.

5. Yang SS, et al. Pediatric isolated oculomotor nerve schwannoma: a new case report and literature review. Pediatr Neurol. 2013;48(4):321–324.

6. Netuka D, Beneš V. Oculomotor nerve schwannoma. Br J Neurosurg. 2003;17(2):168–173.

7. Kawasaki A. Oculomotor nerve schwannoma associated with ophthalmoplegic migraine. Am J Ophthalmol. 1999;128(5):658–660. doi:10.1016/S0002-9394(99)00242-1.

8. Ohata K, Takami T, Goto T, Ishibashi K. Schwannoma of the oculomotor nerve. Neurol India. 2006;54:437–439.

9. Murakami T, Funatsuka M, Komine M, et al. Oculomotor nerve schwannoma mimicking ophthalmoplegic migraine. Neuropediatrics. 2005;36(6):395–398. doi:10.1055/s-2005-873056.

10. Parida S, Sachdeva V, Warkad VU. Isolated cisternal third cranial nerve schwannoma: an insight into a clinico-radiological features. BMJ Case Rep. 2024;17(12):e262958. doi:10.1136/bcr-2024-262958.

11. Niazi W, Boggan JE. Schwannoma of extraocular nerves: survey of literature and case report of an isolated third nerve schwannoma. Skull Base Surg. 1994;4(4):219–226. doi:10.1055/s-2008-1058958.

12. Okamoto S, Handa H, Yamashita J. Neurinoma of the oculomotor nerve. Surg Neurol. 1985;24(3):275–278.

13. Chewning RH, Sasson AD, Jordan LC, Tamargo RJ, Gailloud P. Acute third cranial nerve palsy from a third cranial nerve schwannoma presenting as a saccular aneurysm on three-dimensional computed tomography angiography: case illustration. J Neurosurg. 2008;108(5):1037. doi:10.3171/JNS/2008/108/5/1037.

14. Bentley JN, Prabhu V, Bruner H. Large oculomotor schwannoma presenting as a parasellar mass: case report and literature review. Surg Neurol Int. 2010;1:65.

15. Saetia K, Valavanis A, Mendola C, Schaller K. Schwannoma of the oculomotor nerve: case report and review of the literature. Acta Neurochir (Wien). 2010;152(3):553–559.

16. Headache Classification Committee of the International Headache Society. The International Classification of Headache Disorders, 3rd edition. Cephalalgia. 2018;38(1):1–211. doi:10.1177/0333102417738202.

17. Kim R, Kim JH, Kim E, Yang HK, Hwang JM, Kim JS. Oculomotor nerve tumors masquerading as recurrent painful ophthalmoplegic neuropathy: report of two cases and review of the literature. Cephalalgia. 2015;35(9):825–830. doi:10.1177/0333102414558886.

18. Mrabet S, Nasri A, Kessentini N, Ben Djebara M, Gargouri-Berrechid A, Kacem I, et al. Recurrent painful ophthalmoplegic neuropathy revealing oculomotor nerve schwannoma. Tunis Med. 2021;99(8):919–923.

19. Petruzzelli MG, Margari M, Furente F, Costanza MC, Legrottaglie AR, Dicuonzo F, et al. Recurrent painful ophthalmoplegic neuropathy and oculomotor nerve schwannoma: a pediatric case report with long-term MRI follow-up and literature review. Pain Res Manag. 2019;2019:5392945. doi:10.1155/2019/5392945.
